# Supplementary material for: Development, Establishment, and Validation of a Model for the Mineralization of Periodontium Remodelling Cells: Cementoblasts
Source: Int J Mol Sci. 2023 Sep 7;24(18):13829. doi: 10.3390/ijms241813829 (PMC10531176; doi:10.3390/ijms241813829)
Supplement: Supplementary file 1 [file ijms-24-13829-s001.zip › ijms-2584344-supplementary.pdf]

Supplementary Materials

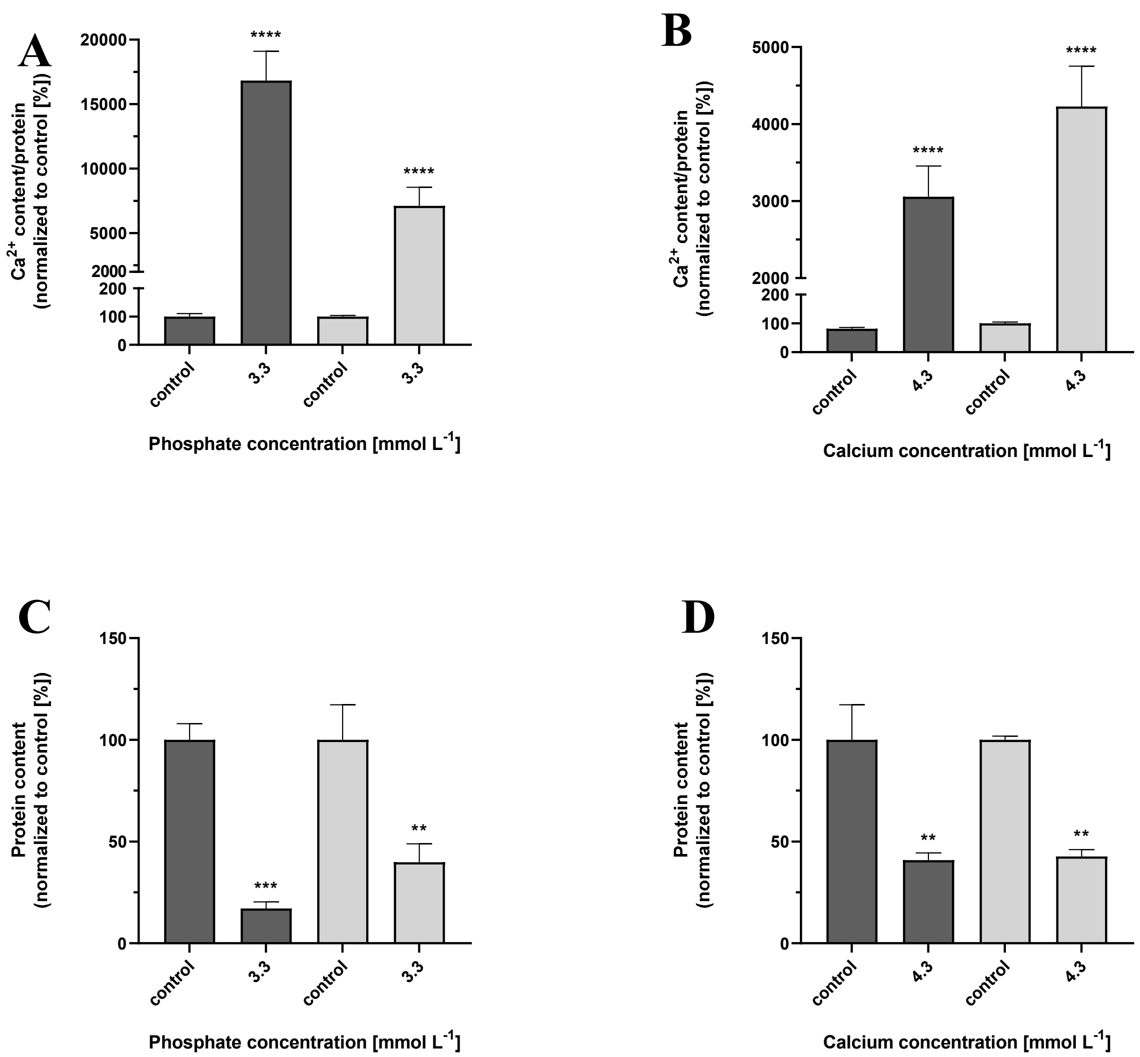

Figure S1. Cementoblast calcify in a concentration-dependent manner in response to high concentration of calcium and phosphate supplementation but show significant decrease in cell viability. Cementoblasts were incubated for three days with (**A,C**) 3.3 mmol L^{-1} phosphate with constant concentrations of 3.8 mmol L^{-1} (dark grey), or 2.3 mmol L^{-1} (light grey) calcium, or (**B,D**) 4.3 mmol L^{-1} calcium with constant concentrations of 2.8 mmol L^{-1} (dark grey), or 2.3 mmol L^{-1} (light grey) phosphate. Data are shown as means ± SEM ($n = 9$). $^{**}P \leq 0.01$, $^{***}P \leq 0.001$ and $^{****}P \leq 0.0001$ compared with the control based on one-way ANOVA. Bonferroni's multiple comparisons were used as a post-test.
